# Genotypic Spectrum in a Cohort of Sri Lankan Patients With Homocystinuria

**DOI:** 10.1002/jmd2.12470

**Published:** 2025-01-21

**Authors:** Hewa Warawitage Dilanthi, Kandana Liyanage Subhashinie Jayasena, Nambage Dona Priyani Dhammika, Neluwa Liyanage Ruwan Indika, Matara Mahavidanage Nishani De Silva, Imalke Kankananarachchi, Pushpa Malkanthi Gardiye Punchihewa, Dharma Irugalbandara, Sabine Schroeder, Kosala Karunaratne, Eresha Jasinge

**Affiliations:** ^1^ Department of Chemical Pathology Lady Ridgeway Hospital for Children Colombo Sri Lanka; ^2^ Department of Biochemistry and Molecular Biology, Faculty of Medicine University of Colombo Sri Lanka; ^3^ Department of Biochemistry, Faculty of Medical Sciences University of Sri Jayewardenepura Nugegoda Sri Lanka; ^4^ Department of Paediatrics, Faculty of Medicine University of Ruhuna Galle Sri Lanka; ^5^ Paediatric Unit Lady Ridgeway Hospital for Children Colombo Sri Lanka; ^6^ Ophthalmology Unit Lady Ridgeway Hospital for Children Colombo Sri Lanka; ^7^ Centogene GmbH Rostock Germany

**Keywords:** betaine, cystathionine beta‐synthase, cystathionine beta‐synthase deficiency, homocystinuria, pyridoxine

## Abstract

Homocystinuria due to cystathionine beta‐synthase (CBS) deficiency is a rare metabolic disorder inherited as an autosomal recessive trait. Spectrum of genetic variants in *CBS* gene and their correlation with the phenotypes of homocystinuria in Sri Lankan patients have not been reported to date. The objective of this study was to identify the genotypes and genotype–phenotype correlations in a cohort of Sri Lankan patients with homocystinuria due to CBS deficiency. We determined the variants in *CBS* gene in 14 Sri Lankan patients with homocystinuria, from 9 unrelated families. The clinical features and the biochemical response to pyridoxine were studied for further correlations. Among the 14 patients, the common clinical features were ectopia lentis (100%), intellectual disability (92%) and marfanoid features (78%) at presentation while three of them had developed osteoporosis (21%). Median age at diagnosis was 8 years (range 2–12). Three pathogenic variants (c.1006C>T, c.785C>T and c.19del) and two likely pathogenic variants (c.869C>T, c.772G>A) in *CBS* gene were identified. Thirteen patients with homozygous genotypes were non‐responsive to pyridoxine while the only patient with the compound heterozygous genotype (c.869C>T/c.772G>A) responded to pyridoxine treatment. The genotypic spectrum observed in Sri Lankan patients is unique and mostly associated with pyridoxine non‐responsiveness. The majority of the patients were identified clinically at a later stage of the disease due to lack of a screening programme in the country. Therefore, it is important to improve the awareness of the disease among the clinicians in the interest of early diagnosis and early commencement of metabolic treatment.

**Summary**

- The genotypic spectrum of homocystinuria due to CBS deficiency in a cohort of Sri Lankan patients who were diagnosed late as having homocystinuria, reveals that the pyridoxine non‐responsiveness is predominant phenotype and highlights the importance of early identification of the disease and commencement of treatment.

## 1 Introduction

Homocystinuria due to cystathionine beta‐synthase [(CBS) (EC 4.2.1.22)] deficiency, also known as classic homocystinuria (OMIM: 236200), inherited as an autosomal recessive trait, is a disorder in the metabolism of sulphur‐containing amino acids. Homocysteine is formed via the transmethylation pathway of the methionine cycle. CBS catalyses the reaction between serine and homocysteine to form cystathionine in the transsulfuration pathway, which leads to the synthesis of cysteine. In CBS deficiency, synthesis of cystathionine from homocysteine is impaired, resulting in elevated levels of homocysteine and methionine in plasma and urine [1].

The incidence of CBS deficiency ranges from 1:20 000 to 1:60 000 in most European countries but it is as low as 1/1 000 000 in some populations [1]. An incidence of 1/6400 is reported in Norway [2], and in Qatar over 1:3000, the highest in the world known so far, attributed to high consanguinity [3]. The incidence of homocystinuria in Sri Lanka is not known.

The metabolic abnormalities in homocystinuria due to CBS deficiency are clinically manifested involving one or several of the four organs/systems: eyes (ectopia lentis, myopia), skeleton (osteoporosis, Marfanoid characteristics), central nervous system (intellectual disability, convulsions and psychiatric abnormalities) and the vascular system (thromboembolism). The spectrum of clinical features of homocystinuria due to CBS deficiency usually manifests in the first or second decade of life [1]. Patients commonly present with ectopia lentis, myopia, intellectual disability and skeletal abnormalities.

Two main phenotypes are identified in these patients as typically milder, but not always, pyridoxine responsive form and a more severe pyridoxine‐nonresponsive form [1]. High dose of pyridoxine is applied in the treatment of CBS as its active form pyridoxal‐5′‐phosphate is the cofactor for CBS. Genetic analysis is not only diagnostic but also assists in distinguishing between these two phenotypes [4].

Human *CBS* gene is located in chromosome 21q22.3 and encodes for a 63‐kDa subunit of 551 amino acid residues [4]. Nearly 200 different disease‐causing genetic variants in the *CBS* gene have been identified [5, 6]. Most of these variants are missense and private [7].

For compound heterozygotes, it is difficult to establish clear genotype/phenotype correlations. For a few variants commonly present in the homozygous state, however, there are well established genotype/phenotype correlations with good concordance between pyridoxine responsiveness and clinical phenotype [5].

We present the genetic analysis performed in patients with homocystinuria due to CBS deficiency in Sri Lanka, referred to a tertiary care hospital. A phenotypic description, including clinical and biochemical data, genotype–phenotype correlations and response to treatment are discussed.

## 2 Methods

This retrospective study includes patients with homocystinuria, referred to the Department of Chemical Pathology, Lady Ridgeway Hospital for Children (LRH), Colombo, Sri Lanka.

Present study was approved by the Ethics Review Committee of the LRH. Data and sample collection procedures were carried out following obtaining the written informed consent of parents/legal guardians. Patients were recruited through contact with physicians caring the patients.

Following inclusion criteria were used to recruit patients who were investigated from 2016 to 2023 and were diagnosed as having homocystinuria; presence of clinical features compatible with homocystinuria, increased total homocysteine (tHcys) and upper normal or high levels of methionine in plasma. A neonatal screening programme for homocystinuria is not available in Sri Lanka. Hence, almost all patients were suspected as having homocystinuria based on clinical grounds and were diagnosed based on investigations.

Plasma tHcys and serum vitamin B12 were measured by automated biochemistry analyser (Cobas c 311) and immunoassay analyser (Abbott ARCHITECT i1000SR) respectively. For tHcys measurement, fasting blood samples were collected in to EDTA tubes on ice and were transported to the laboratory immediately. Plasma was separated within 20 minutes of venepuncture and stored at −20°C until further analyse. Methionine in plasma was measured by HPLC post‐column derivatization system (Pickering Pinnacle PCX HPLC Post‐Column Derivatization). Internal quality control samples, at normal and high levels, were analysed in each run for all tests.

Genetic analysis was performed at CENTOGENE GmbH, Germany using one of the following methods; *CBS* gene sequencing (including next‐generation sequencing [NGS] based copy number variant [CNV] analysis), exome sequencing (CentoXome), or panel Sequencing (CentoMetabolic) (including NGS‐based CNV analysis).

All the identified variants were categorised according to the American College of Medical Genetics and Genomics recommendations [8].

- Class 1—Pathogenic (affects function).
- Class 2—Likely pathogenic (probably affects function).
- Class 3—Variant of uncertain significance (VUS).
- Class 4—Likely benign (probably no functional effect).
- Class 5—Benign (no functional effect).

Pyridoxine responsiveness was the parameter used to evaluate genotype–phenotype relationship. According to the guidelines published in 2017, patients with homocystinuria are classified as pyridoxine responsive if they achieve tHcys levels less than 50 μmol/L while on pyridoxine 10 mg/kg/day up to a maximum of 500 mg/day for 6 weeks. If the reduction in tHcy levels is less than 20% of the baseline, the patient is classified as unresponsive. A reduction in tHcy level more than 20% of the baseline but remaining above 50 μmol/L is classified as partial responsiveness [5]. Majority of the patients in this cohort were diagnosed and were started on pyridoxine before these guidelines were published. Kluijtmans et al. [9] has classified patients with a reduction in plasma tHcys below 50 μmol/L following 6 weeks of pyridoxine treatment, as pyridoxine responsive and all other patients as pyridoxine‐nonresponsive.

At the initial period of investigating these patients, plasma concentrations of tHcys were analysed in a private laboratory. The values were reported as “more than 50 μmol/L” in patients with plasma tHcys levels greater than the upper limit of detection of the assay. Hence, the percentage reduction of plasma homocysteine levels following pyridoxine treatment could not be calculated. During the follow‐up period of these patients only, an analytical method was established in the laboratory at LRH and the exact value of plasma tHcys was reported.

Due to above limitations, pyridoxine responsiveness was defined irrespective of the time of commencement of pyridoxine. Calculation of the percentage of reduction of plasma homocysteine was not possible in some patients and the 50 μmol/L was considered as the cut‐off value to determine the response to pyridoxine.

## 3 Results

The 14 patients included in the study were from nine unrelated families and 10 (71.4%) were male. Five families had two affected siblings in each. Parental consanguinity was reported in 4 families (44.4%). The median age of presentation with symptoms was 6 years (range 5–10). Median age at diagnosis was 8 years (range 2–12). Thirteen patients (92.8%) in the cohort were not responding to pyridoxine. The genotypic and phenotypic characteristics of the patients are summarised in Table 1.

**TABLE 1:** Phenotypic and genotypic characteristics of a cohort of Sri Lankan children with homocystinuria due to CBS deficiency.

Sibship	Patient no.	Gender	Zygosity	Nucleotide change in cDNA for *CBS*	Predicted amino acid changes (effect on coding region)	Response to pyridoxine	Consanguinity	Age at presentation for investigations (years)	Associated phenotypes
1	N1	Female	Hetero	c.869C>T/c.772G>A	p.(Pro290Leu)/ p.(Gly258Ser)	Yes	No	8	Ectopia lentis, myopia, intellectual disability, depression and personality changes, Marfanoid features, change in the colour of hair
2	N2	Male	Homo	c.772G>A/c.772G>A	p.(Gly258Ser)/p.(Gly258Ser)	No	Yes	5	Ectopia lentis, myopia, glaucoma, intellectual disability, Marfanoid features, change in the colour of hair
	N3	Male	Homo	c.772G>A/c.772G>A	p.(Gly258Ser)/p.(Gly258Ser)	No	Yes	Family screening at 2 years	Screened for homocystinuria before symptoms developed since elder brother was a diagnosed patient. Later developed ectopia lentis. Also had intellectual disability, hyperactive behaviour, Marfanoid features.
3	N4	Male	Homo	c.772G>A/c.772G>A	p.(Gly258Ser)/p.(Gly258Ser)	No	No	10	Global development delay, behavioural changes, ectopia lentis, myopia, corneal stromal dystrophy, Marfanoid features, microcephaly, intellectual disability
4	N5	Male	Homo	c.1006C>T/c.1006C>T	p.(Arg336Cys)/p.(Arg336Cys)	No	Yes	7	Ectopia lentis, glaucoma, Marfanoid features
5	N6	Female	Homo	c.1006C>T/c.1006C>T	p.(Arg336Cys)/p.(Arg336Cys)	No	Yes	5	Ectopia lentis, myopia, sero negative poly articular juvenile idiopathic arthritis, personality changes, Marfanoid features, poor academic performance
	N7	Male	Homo	c.1006C>T/c.1006C>T	p.(Arg336Cys)/p.(Arg336Cys)	No	Yes	Family screening at 5 years	Screened for homocystinuria since elder sister was a diagnosed patient before symptoms developed. Later developed ectopia lentis, poor academic performance, Marfanoid features, fatty infiltration of the liver
6	N8	Female	Homo	c.19del/c.19del	p.(Gln7Argfs*75)/p.(Gln7Argfs*75)	No	No	7	Ectopia lentis, myopia, cataract, osteoporosis, Marfanoid features, intellectual disability, behavioural changes
	N9	Male	Homo	c.19del/c.19del	p.(Gln7Argfs*75)/p.(Gln7Argfs*75)	No	No	5	Ectopia lentis, myopia, cataract, osteoporosis, Marfanoid features, intellectual disability
7	N10	Female	Homo	c.19del/c.19del	p.(Gln7Argfs*75)/p.(Gln7Argfs*75)	No	No	6	Ectopia lentis, myopia, glaucoma, Marfanoid features, intellectual disability, aggressive behaviour, mitral valve prolapse, trivial mitral regurgitation
	N11	Male	Homo	c.19del/c.19del	p.(Gln7Argfs*75)/p.(Gln7Argfs*75)	No	No	6	Ectopia lentis, myopia, intellectual disability
8	N12	Male	Homo	c.19del/c.19del	p.(Gln7Argfs*75)/p.(Gln7Argfs*75)	No	No	7	Attention deficit hyperactivity disorder, ectopia lentis, myopia, Marfanoid features
9	N13	Male	Homo	c.785C>T/c.785C>T	p.(Thr262Met)/p.(Thr262Met)	No	Yes	5	Ectopia lentis, intellectual disability, Marfanoid features, osteoporosis
	N14	Male	Homo	c.785C>T/c.785C>T	p.(Thr262Met)/p.(Thr262Met)	No	Yes	Family screening at 2 years	Screened for homocystinuria since elder brother was a diagnosed patient before symptoms developed, ectopia lentis, intellectual disability, later developed Marfanoid features

The plasma tHcys, methionine and serum B12 levels of these patients at the initial stage and plasma tHcys levels during follow‐up are shown in Table 2.

**TABLE 2:** Biochemical characteristics of a cohort of Sri Lankan children with homocystinuria due to CBS deficiency.

	At the time of presentation/initial investigations			Follow‐up	
Patient no.	Plasma homocysteine (μmol/L)	Plasma methionine (6–60 μmol/L)	Vitamin B12 (140–650 pmol/L)	Plasma homocysteine (4.6–8.1 μmol/L)	Other sequelae during follow‐up
N1	> 50 (5.08–15.39)^a^	66.81	291	17	
N2	> 50 (5–15)^a^	69.37	304	299	
N3	282^b^	N/A	364	204	
N4	269^b^	450	173	269	
N5	292 (3.2–10.7)^a^	227	NA	234	
N6	> 50 (5.08–15.39)^a^	91.33	198	248	
N7	> 50 (5.08–15.39)^a^	222.97	222	299	
N8	> 50 (4.4–13.5)^a^	69.41	141	347	Osteoporosis, developed convulsions at the age of 18 years
N9	> 50 (5.46–16.20)^a^	525	145	176	Developed ischaemic heart disease at the age of 23 years
N10	373^b^	79	160	213	
N11	318^b^	177	216	168	
N12	319^b^	57	NA	225	
N13	> 50 (3.36–20.44)^a^	19.67	< 60 (replacement done before further management)	213	Osteoporosis
N14	50 (5.9–16)^a^	NP	< 60 (replacement done before further management)	201	

Abbreviation: NA—not available; NP—not performed.

^a^ The plasma total homocysteine levels within brackets are age related reference ranges provided by the laboratory that analysed the samples.

^b^ Normal reference range: plasma homocysteine 4.6–8.1 μmol/L (at LRH).

Analysis of the coding region of the *CBS* gene allowed the detection of five variants with three recurrent variants. All the patients were homozygous except one patient with a heterozygous variant.

*CBS*, c.1006C>T [p.(Arg336Cys)], *CBS*, c.19del [p.(Gln7Argfs*75)] and *CBS*, c.785C>T [p.(Thr262Met)] are the three pathogenic variants detected in this cohort, and *CBS* c.772G>A [p.(Gly258Ser)] and *CBS*, c.869C>T [p.(Pro290Leu)] were likely pathogenic (Table 3).

**TABLE 3:** Summary of the characteristics of CBS variants detected in the present cohort of Sri Lankan children with homocystinuria.

Nucleotide change in cDNA	Predicted amino acid changes (effect on coding region)	Exon	Variant type	ACMG classification of variants	Frequency in alleles
c.869C>T	p.Pro290Leu	10	Missense	Likely pathogenic	3.6%
c.772G>A	p.Gly258Ser	9	Missense	Likely pathogenic	25%
c.1006C>T	p.Arg336Cys	11	Missense	Pathogenic	21.4%
c.19del	p.Gln7Argfs*75	3	Frameshift	Pathogenic	35.7%
c.785C>T	p.Thr262Met	9	Missense	Pathogenic	14.3%

Abbreviation: ACMG: American College of Medical Genetics.

Interestingly patients with homozygous variants from four families were from the Southern Province of Sri Lanka. Among those families, patients from two families had the same homozygous pathogenic variant (*CBS*, c.1006C>T) and the parents were consanguineous. Patients from other two families also shared a common homozygous pathogenic variant (*CBS*, c.19del) though their parents were non‐consanguineous.

Another two pathogenic homozygous variants were detected in two siblings born to consanguineous parents from Central Province in the country: (*CBS*, c.785C>T) and (*CBS*, c.772G>A).

Only one patient who responded to pyridoxine had two heterozygote variants, with likely pathogenic variants (*CBS*, c.869C>T) and (*CBS*, c.772G>A). Another two siblings were homozygous for the (CBS, c.772G>A) variant. All three patients are from Western Province. Compared to the patient with a heterozygous variant the patients who were homozygous for *CBS*, c.772G>A, have developed clinical features at an early age (Table 1).

Ectopia lentis (100%), intellectual disability (92%) at presentation or developed during the course of the follow‐up and Marfanoid features (78% at presentation) were the most common clinical findings. One patient developed seizures and another experienced ischaemic heart disease during the follow‐up period and they were siblings with the *CBS*, c.19del variant (Table 1). Three patients (21.42%) were screened before development of symptoms; however, during the follow‐up they presented with ophthalmic manifestations and neuro‐psychiatric manifestations.

## 4 Discussion

This study reports the spectrum of genotypes, phenotypes and their correlations for the first time in a cohort of Sri Lankan patients with homocystinuria. Due to the absence of a national newborn screening programme in Sri Lanka, the exact birth incidence of patients with homocystinuria remains undetermined.

The results are in agreement when compared with previously reported data on the correlation between genotype and phenotype.

Most of the variants reported in the *CBS* gene are missense, and the majority of are private mutations [7]. In this cohort, all were missense variant except *CBS*, c.19del which was a frameshift variant. The most frequently encountered variants in other parts of the world, c.833T>C [(p.Ile278Thr)] and c.919G>A [(p.Gly307Ser)] [10, 11, 12, 13], were not detected.

The most frequently encountered pyridoxine‐responsive variant c.833T>C is found in European population [10]. When homozygous to this variant, a pyridoxine responsive mild phenotype is present and are diagnosed at an older age (8–29 years) [10, 11, 12]. The other common variant c.919G>A is pyridoxine‐nonresponsive [10, 13]. It is very frequent in patients of “Celtic” origin and occurs in 71% of Irish alleles [4, 13]. The patients have a severe phenotype, clinically diagnosed at a younger age [10].

Some specific variants have been frequently detected in certain populations, such as the c.919G>A [(p.Gly307Ser)] in the Irish, c.572C>T [(p.Thr191Met)] in Spanish, Portuguese and South Americans and c.1006C>T in the Qatari population [14, 15, 16, 17]. All of these variants have caused severe pyridoxine non‐responsive form when inherited in the homozygous state. Among these frequently encountered variants, only *CBS*, c.1006C>T is detected repeatedly in this studied cohort of patients.

Patient N1 was heterozygous having likely pathogenic variants c.772G>A and c.869C>T, in the *CBS* gene (Table 1). Sperandeo [18] reports a patient from Spain with *CBS*, c.869C>T variant in exon 8 with associated, Hermansky Pudlak syndrome 2 (characterised by oculocutaneous albinism and clotting abnormalities), and pyridoxine responsiveness.

The c.772G>A variant was also found in other three patients in homozygous state (patient N2, N3 and N4) (Table 1). Interestingly, the c.772G>A variant appears to be associated with a range of clinical features, from progressive visual impairment to early developmental delay and microcephaly. This suggests that even within a specific genotype, there can be variability in the phenotype. The heterozygous nature of the c.772G>A and c.869C>T variants in patient N1, who presented with milder symptoms, underscores the phenotypic diversity that can arise from compound heterozygosity in the *CBS* gene.

The c.1006C>T variant in patients N5, N6, N7 was first reported by de Franchis et al. [19] in an English compound heterozygote with another severe variant c.919G>A, who was pyridoxine nonresponsive. A Korean patient was compound heterozygous with c.1058C>T [p.Ser353Leu] and pyridoxine nonresponsive [20]. Same c.1006C>T variant has been identified in patients from Australia and in the Iberian Peninsula including one homozygous Portuguese patients [10, 21]. El‐Said reports this as a common variant in Qatar where 53 patients from tribe M and 3 patients from tribe K were homozygous for c.1006C>T in the *CBS* gene, and all were B6 nonresponsive [3]. This is a known variant that completely removes enzyme activity resulting in pyridoxine non‐responsiveness.

Patients N8, N9, N10, N11 and N 12 had the same c.19del variation, which is pathogenic (Table 1). This variant occurs due to deletion of cytosine at the 19th position of the exon 1 in the coding region of the *CBS* gene. It creates a shift in the reading frame starting at codon 7. The new reading frame ends in a stop codon 74 positions downstream, resulting in a truncated protein with loss of activity. Same variant has been reported in three patients from North India who were compound heterozygotes [22].

The correlation between genotype and phenotype in homocystinuria is complex, as specific mutations in the *CBS* gene can result in a wide spectrum of clinical manifestations. In this cohort, the most common pathogenic variant, c.19del, was associated with a severe phenotype, including early‐onset ocular complications such as cataracts, convulsions and later‐life vascular complications like ischemic heart disease. This variant, which leads to a frameshift and premature truncation of the CBS protein, is consistent with the severe clinical presentation observed. Patients with the homozygous c.1006C>T variant also displayed a severe, pyridoxine nonresponsive phenotype, with two patients from the same family exhibiting significant clinical features and one patient showing commendable academic performance despite the severe genotype. This suggests that while certain genotypes are linked to severe phenotypes, the clinical presentation may still vary.

Kim et al. [23] reports a Norwegian female patient with lens subluxation, Marfanoid features and developmental delay, diagnosed as having homocystinuria at the age of 12 years. She had *CBS* c.785C>T variant reported for the first time and she had no pyridoxine responsiveness. Subsequently the same variant in exon 7 was reported in patients from United States of America with thromboembolic episodes and intellectual disability [24]. In this cohort, patient N13 and N14, who were siblings, also had same homozygous missense variant in exon 9 (c.785C>T) (Table 1). Same variant in homozygous state has been identified in a patient in North India with ocular symptoms, developmental delay, intellectual impairment, neuropsychiatric and behavioural changes [22].

In a North Indian cohort of 14 patients with homocystinuria, Sanger sequencing of the *CBS* gene identified 15 distinct pathogenic or likely pathogenic variants, with compound heterozygosity observed in six patients. In contrast, our cohort of 14 patients exhibited 5 different pathogenic or likely pathogenic variants, with compound heterozygosity found in only one patient [22].

These findings highlight the importance of understanding both the specific genotype and the broader clinical context in managing homocystinuria. While some variants are well‐characterised in Western populations and associated with distinct phenotypes, their absence in this Sri Lankan cohort underscores the need for population‐specific studies to better understand the genotype–phenotype correlations in diverse groups.

In the management of patients with homocystinuria, dietary treatment is considered as a sole treatment or combined with pyridoxine. Initiating dietary restrictions in late diagnosed patients is challenging due to poor compliance, however, it can reduce the risk of developing further complications and can lead to improvement, for example, in seizures and behavioural changes.

As these patients enter young adulthood, adherence to dietary management often declines. This reluctance is likely linked to a late diagnosis, as patients who have consumed a regular diet for years may resist the necessary dietary changes. Consequently, this poor compliance may contribute to the elevated plasma tHcys levels observed during follow‐up. Additionally, as these individuals age, there is a noted tendency for the development of further complications such as osteoporosis, convulsions, and ischemic heart disease.

Currently these patients receive pyridoxine at a dosage of 10 mg/kg/day in two divided doses, not exceeding 500 mg/day. Additionally, they undergo dietary management with methionine restricted diet, receive regular low‐dose folic acid supplements, and have their serum vitamin B12 levels monitored to correct any deficiencies. Each patient is regularly followed‐up depending on their age and the clinical condition.

In conclusion, the spectrum of variants in *CBS* gene observed in Sri Lankan patients is different from that observed in Western countries. This underscores the need for population‐specific studies to better understand the genotype–phenotype correlations in diverse groups. The most common pathogenic variant identified in this cohort, c.19del, accounted for 35.7% of the defective alleles, with a high prevalence of recurrent mutations. These genotypes were predominantly found in patients from the Southern, Western and Central provinces of Sri Lanka. The majority of patients were diagnosed at a later stage of the disease, typically presenting with ocular symptoms, and as they aged, some developed vascular or skeletal complications. These findings underscore the need to raise awareness among clinicians regarding the early identification of patients with homocystinuria. Early detection is crucial, as it can significantly improve the quality of life for patients through timely and appropriate management. The high frequency of pyridoxine non‐responsiveness observed in this cohort further suggests that incorporating betaine as an adjunct treatment could be beneficial in achieving target plasma tHcys levels. The phenotypic diversity observed, calls for personalised management strategies to optimise patient outcomes.

## Author Contributions

**Hewa Warawitage Dilanthi:** data collection, concept and design, analysis and interpretation of data, drafting the article, accountable for all aspects of the work in ensuring that questions related to the accuracy or integrity of any part of the work are appropriately investigated and resolved. **Kandana Liyanage Subhashinie Jayasena:** data collection, interpretation of data, reviewing the manuscript critically for important intellectual content, final approval of the version to be published. **Nambage Dona Priyani Dhammika:** data collection, reviewing the manuscript critically for important intellectual content, final approval of the version to be published. **Neluwa Liyanage Ruwan Indika:** data collection, concept and design, analysis and interpretation of data, reviewing the manuscript critically for important intellectual content, final approval of the version to be published. **Matara Mahavidanage Nishani De Silva:** data collection, analysis and interpretation of data, revising the manuscript, critically for important intellectual content. **Imalke Kankananarachchi:** concept and design, interpretation of data, reviewing the manuscript critically for important intellectual content, final approval of the version to be published. **Pushpa Malkanthi Gardiye Punchihewa:** concept and design, reviewing the manuscript critically for important intellectual content, final approval of the version to be published. **Dharma Irugalbandara:** concept and design, reviewing the manuscript critically for important intellectual content, final approval of the version to be published. **Sabine Schroeder:** genetic testing (analysis), interpretation of data, reviewing the manuscript critically for important intellectual content, final approval of the version to be published. **Kosala Karunaratne:** concept and design, reviewing the manuscript critically for important intellectual content, final approval of the version to be published. **Eresha Jasinge:** data collection, concept and design, interpretation of data, reviewing the manuscript critically for important intellectual content, final approval of the version to be published.

## Disclosure

This article does not contain any studies with human or animal subjects performed by the any of the authors.

## Ethics Statement

Present study was approved by the Ethics Review Committee of the Lady Ridgeway Hospital for Children, Colombo, Sri Lanka.

## Consent

All procedures followed were in accordance with the ethical standards of the responsible committee on human experimentation (institutional—Ethical Review Committee, Lady Ridgeway Hospital for Children, Colombo, Sri Lanka) and with the Helsinki Declaration of 1975, revised in 2000. Written informed consent was obtained from the guardians of all patients for being included in the study. Proof that informed consent was obtained will be available upon request. If doubt exists whether the research was conducted in accordance with the Helsinki Declaration, the authors will explain the rationale for their approach, and demonstrate that the institutional review body explicitly approved the doubtful aspects of the study. Patient identifying information is not included in this article.

## Conflicts of Interest

The authors declare no conflicts of interest.

## Data Availability

The data that support the findings of this study are available from the corresponding author upon reasonable request.
